# Multigenerational inheritance of parasitic stress memory in *Drosophila melanogaster*

**DOI:** 10.1093/eep/dvaf023

**Published:** 2025-09-04

**Authors:** Shagufta Khan, Ravina Saini, Runa Hamid, Rakesh K Mishra

**Affiliations:** CSIR—Centre for Cellular and Molecular Biology, Hyderabad, 500007, India; CSIR—Centre for Cellular and Molecular Biology, Hyderabad, 500007, India; Academy of Scientific and Innovative Research (AcSIR), Ghaziabad, 201002, India; CSIR—Centre for Cellular and Molecular Biology, Hyderabad, 500007, India; Tata Institute for Genetics and Society, Bengaluru, 560065, India; CSIR—Centre for Cellular and Molecular Biology, Hyderabad, 500007, India; Academy of Scientific and Innovative Research (AcSIR), Ghaziabad, 201002, India; Tata Institute for Genetics and Society, Bengaluru, 560065, India

**Keywords:** *Drosophila melanogaster*, *Leptopilina boulardi*, host–parasitoid, parasitic stress, multigenerational epigenetic inheritance, transgenerational epigenetic inheritance, immune priming

## Abstract

Organisms detect harmful environmental conditions and employ strategies to protect themselves. Additionally, they can communicate these experiences to the next generation or beyond through non-DNA sequence-based mechanisms, known as intergenerational or transgenerational epigenetic inheritance, respectively. Using a specialist larval parasitoid, *Leptopilina boulardi*, and its host, *Drosophila melanogaster*, we demonstrate that parental experience of parasitic stress leads to increased survivability in the immediate offspring of the host. Furthermore, we observe that this increased survivability in response to parasitic stress is transmitted transgenerationally when the grandparents, but not the parents, have been exposed to the parasitoid. This increased survivability is primarily inherited through male parents, with one form of effect being enhanced immune priming at the larval stage. Our study suggests that stress exposure during the pre-adult stage of the host provides lifetime benefits for its progeny, enabling them to better cope with future parasitic attacks.

## Introduction

When faced with detrimental environmental conditions, organisms often adopt strategies to enhance the survival of offspring. Since such environmental encounters typically occur over relatively short evolutionary timescales, it is unlikely that changes in DNA sequence alone can provide the rapid adaptive plasticity required to cope with these challenges. In such scenarios, non-genetic or epigenetic mechanisms offer a significant advantage by enabling faster—and often reversible—adaptive responses that can span multiple generations [1–3]. These multigenerational effects include both intergenerational inheritance, where epigenetic changes are observed in the immediate offspring of exposed individuals, and transgenerational inheritance, where effects persist in descendants that were never directly exposed to the initial stimulus.

While epigenetic inheritance in response to abiotic stressors is well documented, responses to biotic interactions, which are equally prevalent, have only recently come to light [4]. These include non-mutualistic interactions between species, such as parasitism. In parasitism, one organism, the parasite or pathogen, causes harm to the other organism, the host, by either living on or inside it [5]. As a result of such interactions, multigenerational epigenetic effects encompassing both behavioural and physiological defences have been reported in a wide range of taxa, including bees [6, 7], pipefish [8–10], ragworms [11], honeycomb moths [12], *Drosophila* [13–15], beetles [16–20], brine shrimp [21], *C. elegans* [22, 23], and mice [24].

The genus *Drosophila* is host to a plethora of parasites in the natural environment, including viruses, bacteria, fungi, and even parasitoid insects [25]. Among them, female parasitoid wasps of the *Leptopilina* genus infect the larval stages of *Drosophila*. They oviposit their eggs into the larval hemocoel, along with immuno-suppressive factors such as venom proteins or virus-like particles [26–28]. In cases of successful infection, the developing wasp consumes the host entirely, develops within the host system, and eventually emerges as an adult from the host pupal case. Occasionally, the *Drosophila* larva mounts a successful immune response, kills the developing wasp, and survives to adulthood. Such flies are referred to as escapee flies [28, 29].

In addition to immune responses like encapsulation, *Drosophila* exhibits numerous other physiological and behavioural defences to safeguard themselves against infection by adult wasps, both at pre-adult and adult stages. For instance, when *Drosophila* adults sense the presence of wasps, they either prefer laying eggs in ethanol- or alkaloid-containing food to medicate their offspring against wasp infection at the larval stage [13, 15, 30–33] or suspend oviposition [34]. Remarkably, the oviposition suspension behaviour is communicated to naïve individuals in an intra- or inter-specific manner to confer protection against infection [34–36]. They also increase the production of recombinant over non-recombinant offspring [37], which may impart fitness to the progeny [38]. Additionally, the host can prime the immune system of their offspring upon cohabitation with adult wasps [14]. At the pre-adult stages, larvae also exhibit rolling behaviour to avoid wasp attacks [39, 40].

A particularly intriguing outcome arising from host–parasitic interactions is immune priming. This phenomenon entails an improved immune response upon re-encountering a pathogen or parasitoid [41, 42]. In the context of *Drosophila’s* association with its parasitoid, *Leptopilina boulardi*, immune priming denotes the *Drosophila* host’s capacity to mount a more robust and effective immune response when facing the same or a similar parasitoid following an initial exposure. This process follows a three-step progression: initial exposure, memory formation, and the heightened response upon re-challenge [14, 43]. Evidence from invertebrates suggests that this primed state can be passed on to offspring via both male and female germlines, encompassing both intergenerational and transgenerational inheritance [4, 42]. Mechanistically, this might involve the activation of immune-related genes, production of antimicrobial peptides, or other mechanisms that assist the host in recognizing and responding more effectively to *L. boulardi* in future encounters [44–46].

Previous studies [47–51] have shown that sustained parasitic pressure can drive the evolution of resistance in genetically diverse *Drosophila* populations through long-term selection—often accompanied by trade-offs in fitness. However, it remains unclear whether parasitic stress over shorter timescales, such as a single generation, can prime the immune system of offspring in the absence of genetic variation or selection. This gap is especially important in understanding the role of non-genetic inheritance mechanisms in host defence.

In this study, we leveraged the specialist host–parasitoid interaction between *D. melanogaster* and *L. boulardi* to investigate whether exposure to parasitism can induce intergenerational and transgenerational immune priming. Using a wild-type (Canton-S) strain of *Drosophila*, we show that the offspring of wasp-experienced parents exhibit increased resistance to parasitic infection compared to the offspring of naïve parents.

We further dissect the contribution of each parent to this inherited resistance. Our findings indicate that enhanced survivability is more robustly transmitted through the paternal lineage, whereas maternal contributions are limited to the immediate generation. Notably, we demonstrate that this effect persists transgenerationally through the male germline.

Finally, we explore the physiological basis of this inheritance and find that larvae born to wasp-experienced fathers exhibit elevated lamellocyte levels upon simulated immune challenge, suggesting a link between inherited resistance and enhanced cellular immune responses. Taken together, our findings suggest a non-genetic mechanism of immune priming that exhibits both intergenerational and transgenerational patterns of inheritance.

## Materials and methods

### Fly strain and culture

In the current study, the wild-type strain of *Drosophila melanogaster*, or fruit fly, named Canton-S (CS), was used. Flies were cultured on a standard medium consisting of corn flour (75 g/l), sugar (80 g/l), yeast (24 g/l), malt (60 g/l), agar (10 g/l), and preservatives such as propionic acid (5 ml/l), methyl-benzoic acid (5 ml/l), and ortho-phosphoric acid (1 ml/l). Fly stocks were maintained in food bottles at a constant average density of 100–150 flies per bottle. However, experiments were conducted in food vials, maintaining a 10:10 male-to-female ratio. All flies were kept at 25°C with a 12-h light-dark cycle.

### Wasp strain and culture

The Lb17 strain of the parasitoid wasp, *L. boulardi*, used in the study was kindly provided by Shubha Govind (Biology Department, The City College of the City University of New York). Wasps were cultured on the *CS* strain of *D. melanogaster*, as previously described [52]. Briefly, 2- to 4-day-old flies were allowed to lay embryos for 48 h at 25°C in standard medium vials. After 48 h, the flies were removed, and 6–8 young, pre-mated female and male wasps were added to the vials to infect the 0- to 48-h-old hosts after egg laying (AEL; second instar fly larvae). Flies that survived the wasp infection (escapee flies) were removed from the vials immediately upon emergence, and vials were kept for further development of the wasps. After 20–22 days, freshly eclosed wasps were collected and used for parasitic stress experiments.

### Multigenerational parasitic stress

**Figure 1.**
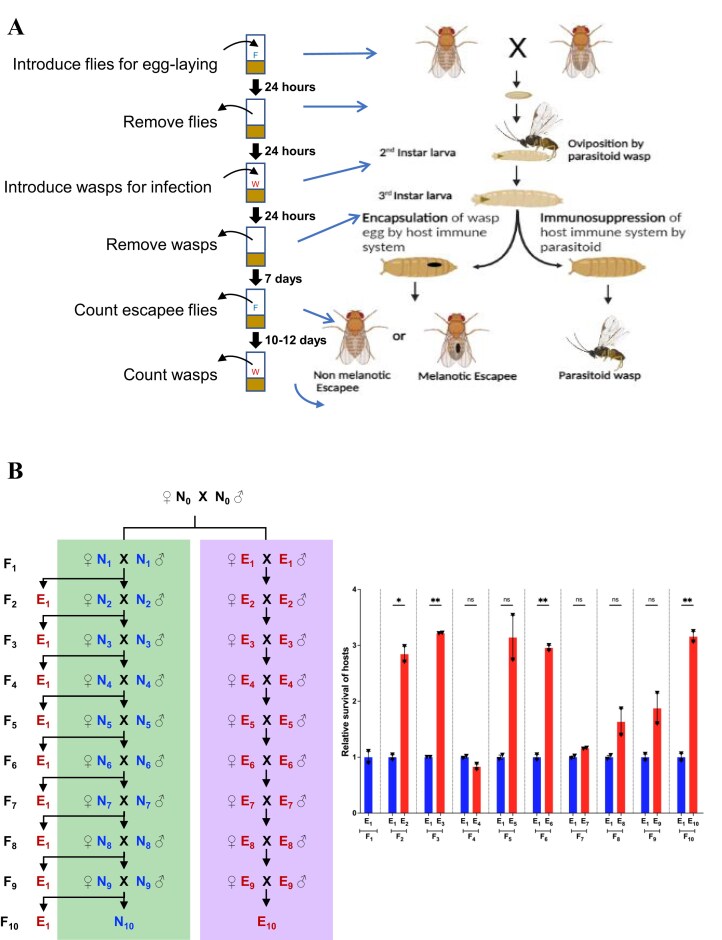
Multigenerational parasitic stress. (A) The parasitoid wasp *Leptopilina boulardi* lays its eggs in the second instar larvae of *Drosophila melanogaster*, along with immune-suppressive factors. The host’s initial response is to activate cellular immunity, encapsulating the wasp egg with specialized immune cells called lamellocytes. Hosts that successfully encapsulate the egg develop into adults (10 days after egg-laying) and may emerge with or without a black melanotic capsule. If the host fails to encapsulate the egg and the parasitoid effectively suppresses the host’s immune system, the parasitoid emerges 18–20 days post-infection. Adult flies that survive this host–parasitoid interaction are referred to as experienced escapees (W, wasps; F, flies). (B) The mating scheme for multigenerational parasitic stress over 10 generations is illustrated on the left. The lavender box represents the experienced (E) group, where progeny were exposed to parasitic stress at the second instar larval stage in each generation. The green box represents the naïve (N) group, in which subsequent generations were reared without parasitic stress, though some were exposed in each generation to assess survival upon naïve exposure. The experiment was conducted in two biological replicates. The number of escapees was recorded in each generation to calculate survival rates. The bar graph on the right presents the mean relative survival ±SEM, with individual replicates shown as triangles (see Supplementary Table S1). Red and blue fonts indicate parasitic stress and no stress, respectively, with subscripts denoting the generation. Red bars represent the relative survival of the experienced group, while blue bars show the survival of the naïve group. Significance levels are indicated as follows: ** for *P* < .01, * for *P* < .05, and ns for non-significant results (*P* > .05).

**Figure 2.**
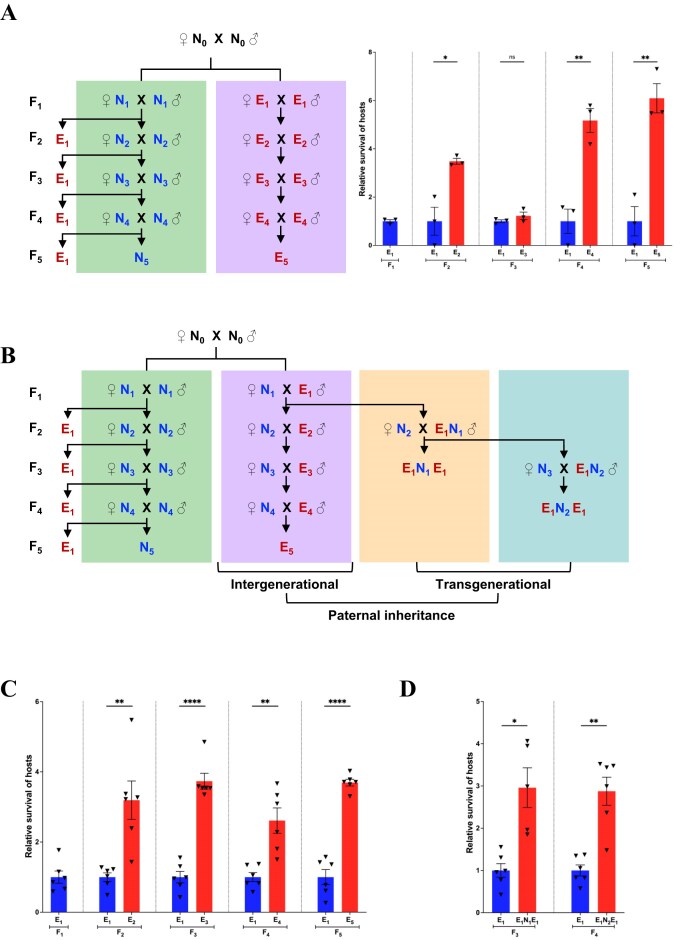
Paternal contribution to the parasitic stress resistance. (A) The mating scheme for multigenerational parasitic stress over five generations is shown on the left. The lavender box represents the experienced (E) group, where progeny were exposed to parasitic stress at the second instar larval stage in each generation. The green box represents the naïve (N) group, in which subsequent generations were reared without parasitic stress, though some were exposed in each generation to assess survival upon naïve exposure. The experiment was conducted in three biological replicates. The number of escapees was recorded in each generation to calculate survival rates. The bar graph on the right presents the mean relative survival ±SEM, with individual replicates shown as triangles (see Supplementary Table S2). Red and blue fonts indicate parasitic stress and no stress, respectively, with subscripts denoting the generation. Red bars represent the relative survival of the experienced group, while blue bars show the survival of the naïve group. Significance levels are indicated as follows: ** for *P* < .01, * for *P* < .05, and ns for non-significant results (*P* > .05). (B) The mating scheme to investigate paternal contribution and assess the transgenerational nature of parasitic stress memory is illustrated. The lavender box represents the experienced (E) group, where progeny from escapee fathers and naïve mothers were exposed to parasitic stress at the second instar larval stage in each generation. The green box represents the naïve (N) group, where subsequent generations were reared without parasitic stress, though some were exposed in each generation to assess survival upon naïve exposure. The orange and teal boxes represent treatment groups in which one (E_1_N_1_) and two (E_1_N_2_) generations, respectively, were stress-free after initial exposure (E_1_) before testing for survival advantage in a subsequent generation (E_1_N_1_E_1_ and E_1_N_2_E_1_). The experiment was conducted in six biological replicates. The number of escapees was recorded in each generation to calculate survival rates. (C) and (D) bar graphs show the mean relative survival ±SEM corresponding to the mating scheme in (B), with individual replicates represented by triangles (see Supplementary Table S3). Red and blue fonts indicate exposure to parasitic stress and no stress, respectively, with subscripts denoting the generation of treatment. Red bars represent the relative survival of the experienced group, while the blue bar shows the relative survival of the naïve group. Significance levels are indicated as follows: **** for *P* < .0001, ** for *P* < .01, * for *P* < .05, and ns for non-significant (*P* > .05).

F_0_  *CS* flies (N_0_) were mated to collect 0- to 24-h F_1_ embryos in food vials. At 24–48 h AEL, the larvae were exposed to parasitic stress by infecting them with 10 female and 10 male Lb17 wasps for 24 h at 25°C. After infection, the wasps were removed, and the infected larvae were allowed to grow until escapee flies and wasps emerged (Fig. 1A). The F_1_ escapee flies, referred to as E_1_ due to their initial exposure, were collected and mated to obtain the 0- to 24-h F_2_ embryos. The F_2_ embryos were then infected again at the second instar larval stage and allowed to grow until F_2_ escapee flies (E_2_) and wasps emerged. This process was repeated for ten generations in two biological replicates (Fig. 1B) and for five generations in three biological replicates (Fig. 2A) to establish the experienced treatment group. The number of escapees with and without melanized wasp eggs, referred to as melanotic capsules, was recorded in each generation. Escapee flies with melanotic capsules were used to set up crosses to assess the multigenerational inheritance of parasitic stress memory.

In parallel, unstressed sibling F_1_ embryos (0–24 h) were collected from F_0_  *CS* flies (N_0_) in food vials and allowed to grow into adult flies without any stress (N_1_). The N_1_ flies were then mated to produce F_2_ embryos. Some of the F_2_ embryos were allowed to grow to adulthood (N_2_) in the absence of stress. However, some batches of 0- to 24-h F_2_ embryos were infected at the second instar larval stage, following the same procedure as the experienced treatment group, to determine the survival rate of naïve hosts in response to parasitic stress. This method of embryo collection to obtain subsequent unstressed generations and exposure of some batches to parasitic stress was performed for ten generations (Fig. 1B) and for five generations (Fig. 2A), establishing the naïve treatment group. Our protocol for parasitic stress follows the standardised experimental conditions, but consideration of a blinded experiment for future studies can be helpful in strengthening the reproducibility of the data.

### Parental contribution

For assessing parental contribution, we exposed F_1_ second-instar *Drosophila* larvae to parasitoids and carefully separated the resulting virgin females and males (E_1_) that managed to escape. We then mated the E_1_ males with naïve virgin females to establish the paternal lineage and E_1_ females with naïve males to establish the maternal lineage. Embryos were collected for 24 h from these crosses and were subjected to wasp infection at 24–48 h AEL, using 10 females and 10 males for infection for 24 h. After infection, larvae were allowed to grow until they emerged as escapee flies (E_2_) or adult wasps (see schematics in Fig. 2B and Fig. S1A). The number of escapees with and without melanotic capsules was recorded in each generation. Only melanotic capsule-containing male and female escapee flies were used for setting up crosses to assess the paternal and maternal contributions of parasitic stress memory to the next generation, respectively. This process was repeated for five consecutive generations (E_1_ to E_5_). The experiment was performed in six biological replicates. In parallel, a naïve treatment group was established as a control for five generations, following the same procedure outlined in the multigenerational parasitic stress section.

### Transgenerational parasitic stress

To evaluate the transgenerational effect, once-exposed (E_1_) male escapee flies were mated with naïve virgin females to establish the paternal lineage, while once-exposed female escapee flies were mated with naïve males to establish the maternal lineage. The resulting F_2_ embryos from these matings were allowed to develop into adults without additional stress. These adult flies are referred to as E_1_N_1_. The E_1_N_1_ males (paternal lineage) and E_1_N_1_ females (maternal lineage) were then mated with naïve virgin females and males, respectively (see schematics in Fig. 2B and Fig. S1A). The F_3_ embryos were collected for 24 h, and 48 h AEL were infected by 10 female and 10 male wasps for 24 h. The number of escapees with and without melanotic capsules was recorded. This process was repeated once more as outlined in the schematic. Only the melanotic capsule-containing male and female escapee flies were used for further crosses. The experiment was performed in six biological replicates.

### Fecundity assay

For the fecundity assay, single-pair matings were performed, and eggs were collected for 12 h each day over a 3-day period. The experiment was conducted in six biological replicates. The total number of eggs laid, representing fecundity, was divided by the number of days, and the average fecundity was calculated for further analysis.

### Immune induction and immunostaining

Immune induction was done as described previously [14]. In brief, the second instar larvae from naïve parents and exposed parents were poked by a sterile needle at their posterior region in 1X PBS and were then transferred to fresh food vials [1–3]. After 24 h, larvae were scooped out of the food vials, washed twice with 1X PBS to remove food remnants, and once with 70% ethanol for surface sterilization. The larvae were then transferred to ice-cold 1X PBS until dissection. Haemolymph from a single larva per well was collected and allowed to settle at the bottom of a 4 mm well slide. Cells were fixed with 4% formaldehyde for 20 min, followed by three washes with 1X PBS-T (0.5% Triton-X) for 5 min each. After blocking in 1% BSA, cells were stained using the primary antibody anti-myospheroid (DSHB #CF.6G11, 1:500 dilution), a lamellocyte marker, since lamellocytes appear after an immune challenge. After washing three times with 1X PBS-T (0.3% Triton-X), cells were incubated with Alexa Fluor® 647 AffiniPure goat anti-mouse IgG secondary antibody (115-605-003, 1:1000 dilution). Finally, the cells were washed three times with 1X PBS-T (0.3% Triton-X) and stained with DAPI for nuclei visualization.

Representative images were taken with an LSM 880 using a 40X oil immersion lens. Each well was scanned for cell counting using an Olympus FV3000 confocal microscope with a 60X oil immersion lens. For the quantification of lamellocytes, the entire well was scanned, and images were processed using ImageJ Version 1.53c (Fiji). DAPI was used to stain total cells, and anti-myospheroid was used to stain lamellocytes, allowing for cell counting in ImageJ. The experiment was conducted in three biological replicates.

### Statistical analysis

The survival of hosts was calculated as a percentage of escapees by dividing the number of escapee flies emerging after parasitic stress by the total number of individuals (larvae/pupae) exposed (see Tables S1–S4). To account for baseline differences or fluctuations across generations, normalized (relative) survival rates were determined by dividing each data point by the mean of the control group within the same generation. These normalized survival rates are presented as bar graphs to visually compare the treatment group to the control group within each generation and were further used to assess statistical significance. Since the naïve group of a specific generation serves as the most appropriate control for the corresponding treatment group, effectively accounting for generational fluctuations, survival advantage was assessed by comparing the naïve group and the experienced group within the same generation.

For the fecundity assay, the mean fecundity was used to generate bar graphs and perform statistical analysis. For immune induction, the number of myospheroid-positive cells was divided by the total number of haemocytes to calculate the percentage of lamellocytes, and the mean percentage was used to generate bar graphs and assess statistical significance.

In all experiments, the Shapiro–Wilk test was conducted to assess normality. Groups that passed the normality test were analysed using a parametric unpaired *t*-test with Welch’s correction, given that there were six or fewer biological replicates. Comparisons with adjusted *P*-values <.05 were considered significant and denoted as follows: ****/#### for *P* < .0001, ***/### for *P* < .001, **/## for *P* < .01, */# for *P* < .05, and ns for non-significant results of *P* > .05. GraphPad 9 software was used to generate bar graphs and perform statistical analysis.

## Results

### Effect of multigenerational parasitic stress on host survival

To assess the effect of multigenerational parasitic stress on host survival, we designed experiments where fly larvae were repeatedly exposed to wasps for 10 generations in two biological replicates (E_1_ to E_10_) (Fig. 1B, Table S1) and five generations in three biological replicates (E_1_ to E_5_) (Fig. 2A, Table S2). This created an experienced treatment group with a history of infection. Conversely, larvae in the naïve treatment group were newly exposed to wasp infection in each generation for ten generations (N_1_ to N_10_) and five generations (N_1_ to N_5_). We considered all flies that emerged after infection, with and without melanotic capsules, to calculate the host survival rate. Flies without melanotic capsules were included for two reasons. First, fly larvae can escape wasp infection not only through encapsulation of the wasp egg (physiological defences) but also by employing a rolling strategy (behavioural defences) [39, 40]. Second, it has been shown that melanization rates in *D. melanogaster* are low [53], which can be attributed to the low haemocyte load compared to other *Drosophila* species [54]. Therefore, the absence of a melanotic capsule does not necessarily indicate a lack of infection. Importantly, only flies that developed visible melanotic capsules—indicating a confirmed infection—were selected as parents for subsequent generations in the experiments.

We observed that infecting the progeny of F_1_ escapee flies (E_1_) at the larval stage i.e. E_2_, resulted in a significant increase in survival after infection (Figs. 1B and 2A, Tables S1 and S2) when compared to the survival after infection of progeny of F_1_ naïve flies (N_1_) at the larval stage. A similar increase in survival was also observed in the subsequent generations when the progeny of the experienced group were infected as compared to the naïve group. Although we observe some fluctuations in host survival across our multigenerational parasitic stress experiments, our results indicate that the progeny of parents exposed to parasitic stress exhibit enhanced resistance and improved survival upon subsequent attacks.

### Male parents effectively pass resistance to parasitic stress to their progeny

We investigated whether both parents equally contribute survival advantage to the progeny by allowing either the male or female escapee flies to give rise to the subsequent generation and thereby contribute to the parasitic stress resistance. Exposed male flies (E_1_) were mated with naïve female flies to examine the paternal inheritance (Fig. 2B). On the other hand, the exposed female flies (E_1_) were mated with naïve male flies to examine maternal inheritance (Fig. S1A). The experiment was carried out for five generations. As a control, naïve parents were taken. Interestingly, transmission of survival advantage to progeny was better through escapee males than through escapee females (Figs. 2C, Table S3), and transmission through escapee females did not extend beyond the immediate next generation (Fig. S2B, Table S4). While progeny from the experienced mother showed better survival in only one generation compared to the once-exposed control, the survival advantage was more than two-fold in the progeny of experienced fathers in the subsequent generations upon repeated exposure.

We further examined if increased survival is a result of an increase in egg lay, such as when a stressed adult female fly tends to lay more eggs once the stress is removed. We checked the fecundity of progeny from stressed parents and found no significant difference in the fecundity of the progeny from stressed parents compared to the naïve flies (Fig. S2, Table S5). These results indicate differences in the perception of and response to the parasitic stress of male and female flies.

### Resistance to parasitic stress is transgenerational

We further explored whether resistance to the parasitic stress is transgenerationally inherited. We set two groups of experiments where in one group, E_1_ males were mated with naïve virgin females to obtain the paternal lineage, and in the other group, E_1_ virgin females were mated with naïve males to obtain the maternal lineage. Progeny from both lineages were collected without any wasp exposure to get unexposed male or female (E_1_N_1_) progeny. E_1_N_1_ males from paternal lineage and E_1_N_1_ females from maternal lineage were then mated with naïve females and males, respectively, and their progeny were exposed to wasps at the second instar larval stage (Fig. 2B and Fig. S1A). These larvae, named E_1_N_1_E_1_ (grandchildren of once-exposed males or females), were allowed to grow in standard conditions. Unlike females, males were more successful in transmitting the increased survival relative to the control to their grandchildren (Fig. 2D and Fig. S1C, Tables S3 and S4). Moreover, we observed that the effect was inherited paternally beyond two generations (E_1_N_2_E_1_). Together, these findings indicate transgenerational inheritance of resistance to parasitic stress via the male germline.

### Parasitic stress resistance is inherited as enhanced cellular immunity

**Figure 3.**
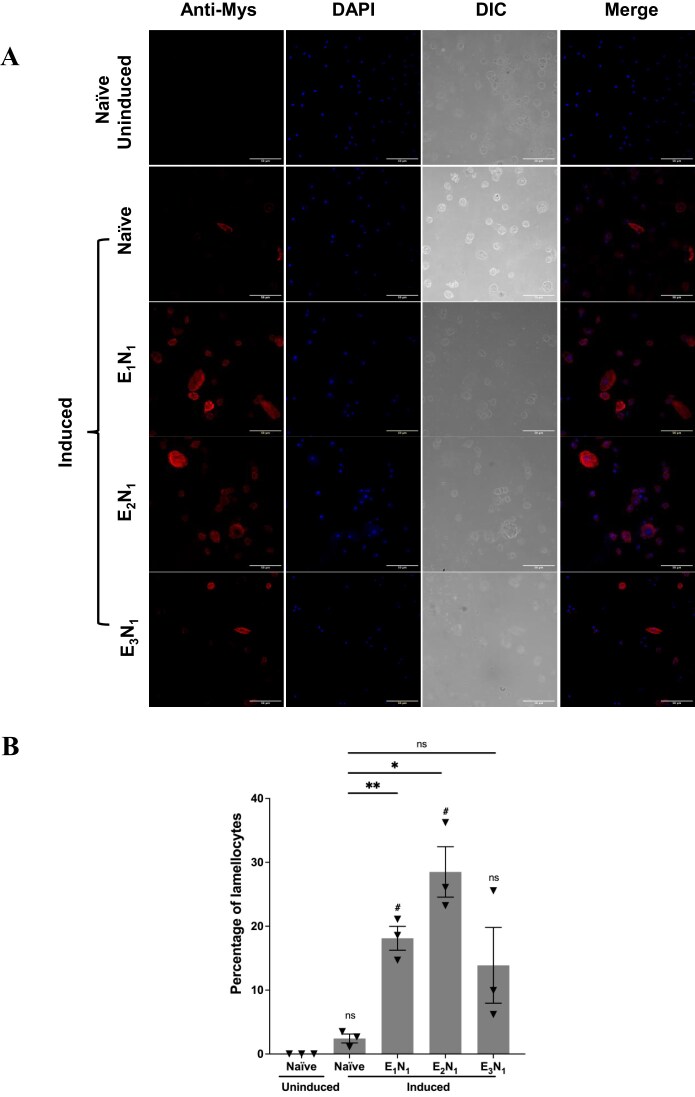
Cellular immune response to parasitic stress in the progeny of male parents. (A) The panels depict images of circulatory haemolymph in third instar larvae from experienced male parents. In the first panel, haemocytes from naïve third instar larvae are shown, stained with anti-myospheroid (red) as a lamellocyte marker and DAPI (blue) for nuclei staining. The second panel shows haemocytes from third instar larvae that were mechanically induced to mimic a wasp attack at the second instar stage. The third panel presents haemocytes from induced larvae (E_1_N_1_) of an experienced male parent. The fourth panel shows haemocytes from induced larvae (E_2_N_1_) of an experienced male parent whose own father was also exposed to parasitic stress. The fifth panel represents haemocytes from induced larvae (E_3_N_1_) of an experienced male parent, whose own father and grandfather were exposed to parasitic stress. Myospheroid-positive cells appear as fully developed lamellocytes (large and elongated) or as precursors committed to lamellocyte development, reflecting an enhanced cellular immune response. Scale bar is 50 μm. (B) The percentage of lamellocytes in the larval haemolymph, as shown in the panels in (A), is presented here (see Supplementary Table S6). The experiment was performed in three biological replicates, with error bars indicating the standard error of the mean (SEM). Significance levels are represented as follows: **/## for *P* < .01, */# for *P* < .05, and ns for non-significant results (*P* > .05). Hashtags denote *P*-values comparing naïve uninduced larvae with the rest of the treatments, while asterisks represent *P*-values comparing naïve induced larvae with the other treatments.

*Drosophila* exhibits a cellular immune response upon wasp attack. It possesses three types of haemocytes engaged in the immune response: plasmatocytes, which constitute 95% of the total haemocytes and eliminate pathogens and injured cells; crystal cells (5%); and lamellocytes, which are rarely observed in healthy larvae. Lamellocytes emerge following plasmatocyte differentiation in response to any foreign immune challenge and are deployed to encapsulate the pathogen, depriving it of oxygen and nutrients [41, 44, 55, 56]. Therefore, we speculated that the survival advantage or resistance observed in the progeny of stressed parents is due to an enhanced cellular immune response. In order to test that, we induced the progeny of exposed parents using a sterile needle to mimic the wasp attack and measured the total number of haemocytes and lamellocytes 24 h post-induction. We observed that the progeny of exposed males exhibited an elevated number of lamellocytes (Mys⁺) as compared to controls (induced larvae from unexposed parents) (Fig. 3, Table S6). A single generation of wasp exposure (E_1_N_1_) resulted in a nearly seven-fold increase in lamellocyte percentage—18% of total haemocytes—compared to naïve-induced larvae (2.4%). Similarly, larvae derived from males exposed for two consecutive generations (E_2_N_1_) showed a further significant increase, with lamellocytes comprising 28.5% of total haemocytes. Although the increase in lamellocyte percentage was not statistically significant in larvae from the E_3_N_1_ group (13.8%), it still reflected an upward trend relative to the control. In contrast, the progeny of the exposed females did not show a significant change in the lamellocyte number compared to the control (Fig. S3, Table S6). These results indicate that the resistance observed after multiple generations of parasitic stress through the male parent is correlated with the enhanced cellular immune response of the hosts.

## Discussion

Organisms are constantly engaged in an evolutionary arms race, especially in host–parasite interactions, where host survival depends on the effectiveness of its defence mechanisms and the parasite’s ability to evade them. In insects, innate immunity serves as a crucial barrier against parasites and pathogens, particularly during embryonic and larval development. If offspring are pre-armed with enhanced immune responses due to epigenetic changes inherited from parents exposed to infection, they may gain a significant survival advantage. This phenomenon—known as immune priming—occurs when parental exposure to biotic stress leads to improved resistance in offspring [4].

In this study, we show that *D. melanogaster* subjected to repeated parasitic stress from *L. boulardi* produce progeny with enhanced resilience against wasp infection. This enhanced survivability is likely mediated by heightened cellular immune responses, particularly through increased differentiation of lamellocytes, specialized immune cells critical for encapsulating wasp eggs. These findings highlight a non-genetic mode of inheritance that facilitates faster and potentially reversible adaptive responses across generations.

Previous work has shown that cohabitation with parasitoid wasps induces intergenerational immune priming in fruit fly offspring via maternal experience [14]. Our findings extend this concept by demonstrating that direct infection—not just social or environmental exposure—can induce heritable immune priming. Importantly, we dissected the parental contribution and found that this memory is transmitted primarily through the male germline, with both intergenerational and transgenerational effects (Fig. 2C and D). In contrast, female parents failed to transmit this memory beyond the immediate generation (Fig. S1 and S3), suggesting sex-specific routes of information transmission.

Interestingly, despite prior findings showing oviposition suppression in female flies exposed to wasps, often via apoptosis in ovarian tissues [34], our fecundity assay revealed no changes in oviposition rate in either male or female escapees after a single exposure (Fig. S2). However, we did not assess the impact of repeated multigenerational exposure on fecundity. Thus, the inability of females to transmit immune priming across generations may reflect sex-specific physiological constraints or alternative stress coping strategies.

Our data show that increased lamellocyte numbers in the progeny of challenged males (Fig. 3) correspond with enhanced survival, supporting the idea that parasitic stress resistance is transmitted as augmented cellular immunity. However, we observe a slight decrease in lamellocyte number in the E_3_N_1_ generation, which may indicate a negative impact of successive wasp exposures. This trend aligns with the modest decline in wasp resistance observed at the fourth generation (E_4_) in Fig. 2C. Such a pattern could reflect the costs of sustained immune activation—constitutive cellular immunity may impose physiological trade-offs that lead to variability in resistance or immune response. We speculate that the reduced lamellocyte levels may result from immune pathway exhaustion or the activation of negative feedback mechanisms over successive generations [49, 57, 58]. Further experiments will be necessary to clarify the underlying cause of this trend. Overall, this transgenerational immune priming suggests that epigenetic modifications in the male germline—possibly in sperm—encode and convey past parasitic experiences to future generations, preparing them for similar threats even in the absence of direct exposure.

Although our study demonstrates that even a single generation of parasitic stress can induce inheritable immune priming—likely through epigenetic mechanisms—the precise molecular nature of these changes remains to be elucidated. Future work should explore whether this inheritance is mediated by non-coding RNAs, histone modifications, or DNA methylation in sperm. It is also important to determine whether these epigenetic responses are specific to parasitic stress or represent a broader, generalized mechanism for coping with environmental challenges. Furthermore, the prevalence and functional significance of such germline-mediated responses across different taxa remain open questions. Understanding these mechanisms could reveal how paternal experiences shape offspring immunity and adaptation, and our findings provide a foundational model for studying the non-genetic inheritance of biotic stress-induced traits.

While our research provides valuable insights into the multigenerational memory transmission of parasitoid stress in *Drosophila*, certain limitations should be taken into consideration for future directions. For example, the study cannot exclude the possibility of inefficient infection in the progeny of experienced parents. It is important to subtract the proportion of uninfected animals from the experimental protocol. It can be done by precise documentation of infected animals with confirmed immune markers [59, 60]. Future experiments incorporating infection efficiency measurements would provide greater precision in interpreting immune outcomes. Furthermore, our interpretation of immune memory is based on cellular quantification. This can be supplemented with profiling of immune marker genes in experienced individuals to track the memory transmission at the molecular level. Taken together, our finding provides a robust platform to investigate transgenerational epigenetic inheritance of biotic stress. In the complex biological world where highly dynamic interspecies interaction can have profound impact on survival, evolution of epigenetic or reversible inheritance processes can have a critical role. Further genetic and molecular investigations will be needed for better understanding and future explorations in this emerging domain of biology.

## Supplementary Material

dvaf023_Supplemental_Files

## Data Availability

All the data are provided in the supplementary tables.
