# Supplementary material for: Multigenerational inheritance of parasitic stress memory in Drosophila melanogaster
Source: Environ Epigenet. 2025 Sep 4;11(1):dvaf023. doi: 10.1093/eep/dvaf023 (PMC12418946; doi:10.1093/eep/dvaf023)
Supplement: dvaf023_Supplemental_Files [file dvaf023_supplemental_files.zip › Figure S1.pdf]

Supplementary Figure S1

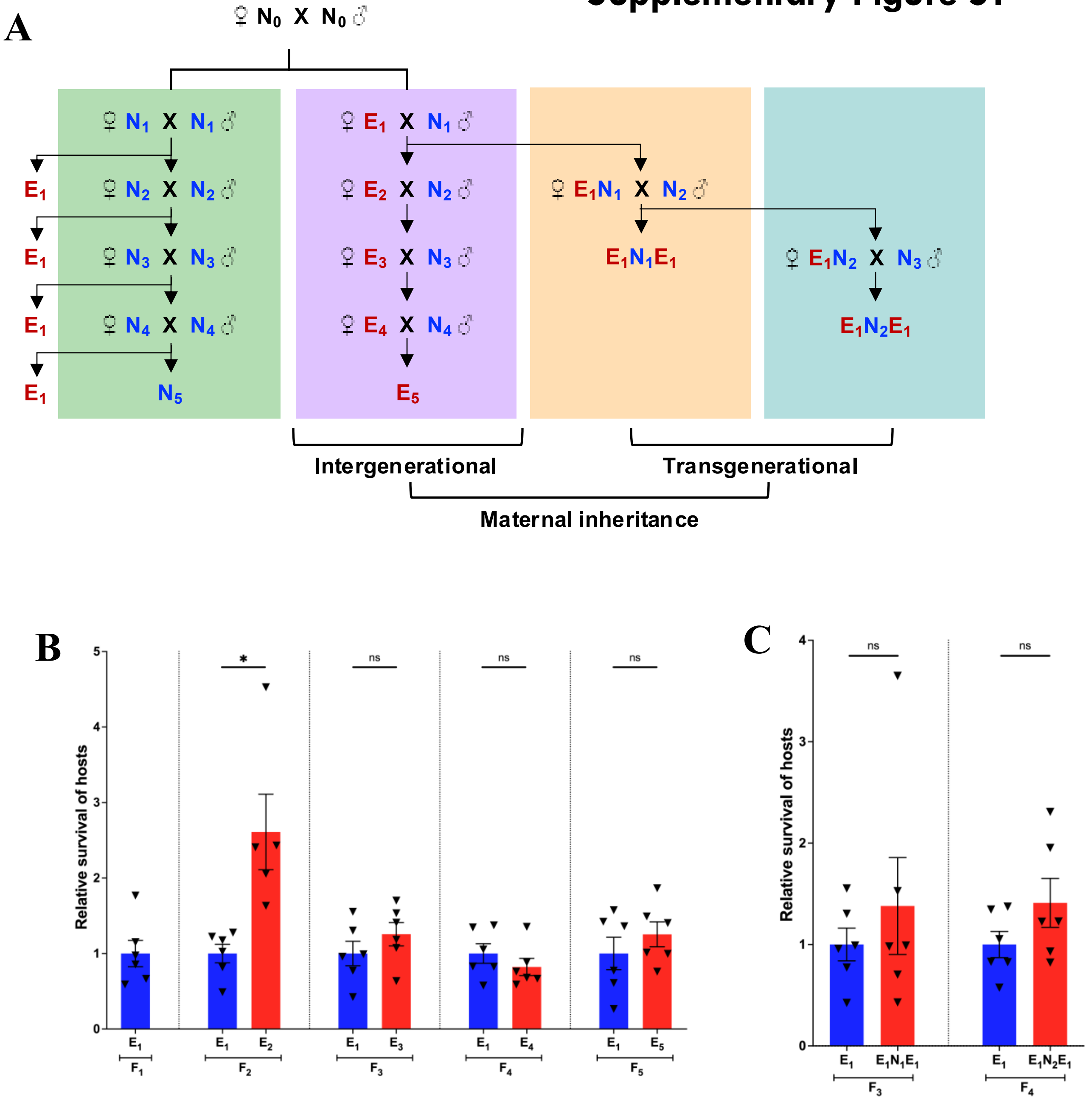

Figure S1. Maternal contribution to the parasitic stress memory. (A) The mating scheme to investigate maternal contribution and assess transgenerational nature of the parasitic stress memory is shown. The lavender box represents the experienced (E) group, where progeny from escapee mothers and naïve fathers were exposed to parasitic stress at the second instar larval stage in each generation. The green box represents the naïve (N) group, where subsequent generations were reared without parasitic stress, though some were exposed in each generation to assess survival upon naïve exposure. The orange and teal boxes represent treatment groups in which one (E_1N_1) and two (E_1N_2) generations, respectively, were stress-free after initial exposure (E_1) before testing for survival advantage in a subsequent generation ($E_1N_1E_1$ and $E_1N_2E_1$). The experiment was conducted in six biological replicates. The number of escapees was recorded in each generation to calculate survival rates. **(B)** and **(C)** bar graphs show the mean relative survival ± SEM corresponding to the mating scheme in (A), with individual replicates represented by triangles (see Supplementary Table S4). Red and blue fonts indicate exposure to parasitic stress and no stress, respectively, with subscripts denoting the generation of treatment. Red bars represent the relative survival of the experienced group, while the blue bar shows the relative survival of the naïve group. Significance levels are indicated as follows: * for $p < 0.05$ and ns for non-significant ($p > 0.05$).
