# Supplementary material for: Multigenerational inheritance of parasitic stress memory in Drosophila melanogaster
Source: Environ Epigenet. 2025 Sep 4;11(1):dvaf023. doi: 10.1093/eep/dvaf023 (PMC12418946; doi:10.1093/eep/dvaf023)
Supplement: dvaf023_Supplemental_Files [file dvaf023_supplemental_files.zip › Figure S2.pdf]

Supplementary Figure S2

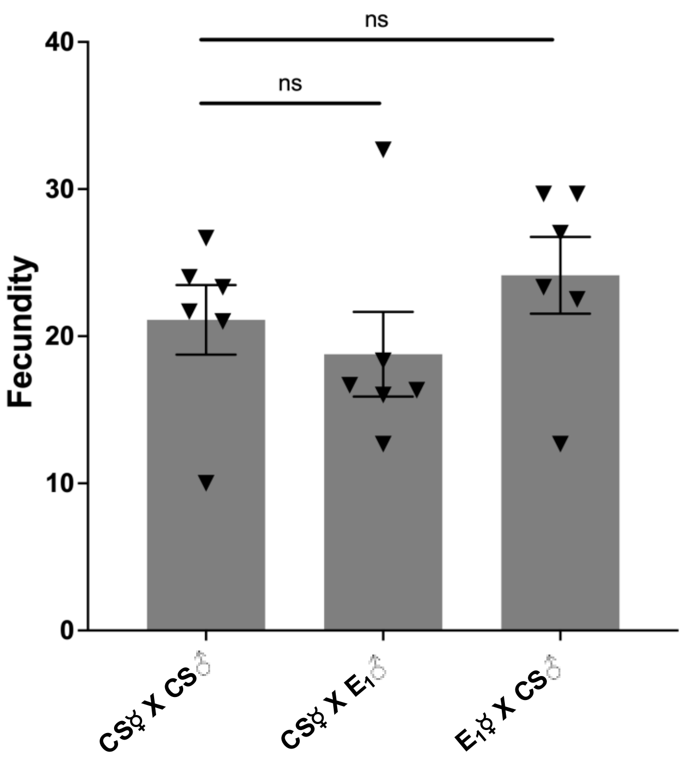

Figure S2. Effect of parasitic stress on fecundity. The number of eggs laid over three days in three groups — control (CS♀ × CS♂), experienced males mated with naïve females (CS♀ × E_1♂), and experienced females mated with naïve males (E_1♀ × CS♂) — was recorded. Fecundity was calculated by dividing the total number of eggs by the number of days, and the mean values are presented in the bar graph. The experiment was performed in six biological replicates, with error bars representing the standard error of the mean (SEM). Significance is denoted as ns for non-significant results ($p > 0.05$).
