# Supplementary material for: Multigenerational inheritance of parasitic stress memory in Drosophila melanogaster
Source: Environ Epigenet. 2025 Sep 4;11(1):dvaf023. doi: 10.1093/eep/dvaf023 (PMC12418946; doi:10.1093/eep/dvaf023)
Supplement: dvaf023_Supplemental_Files [file dvaf023_supplemental_files.zip › Figure S3.pdf]

Supplementary Figure S3

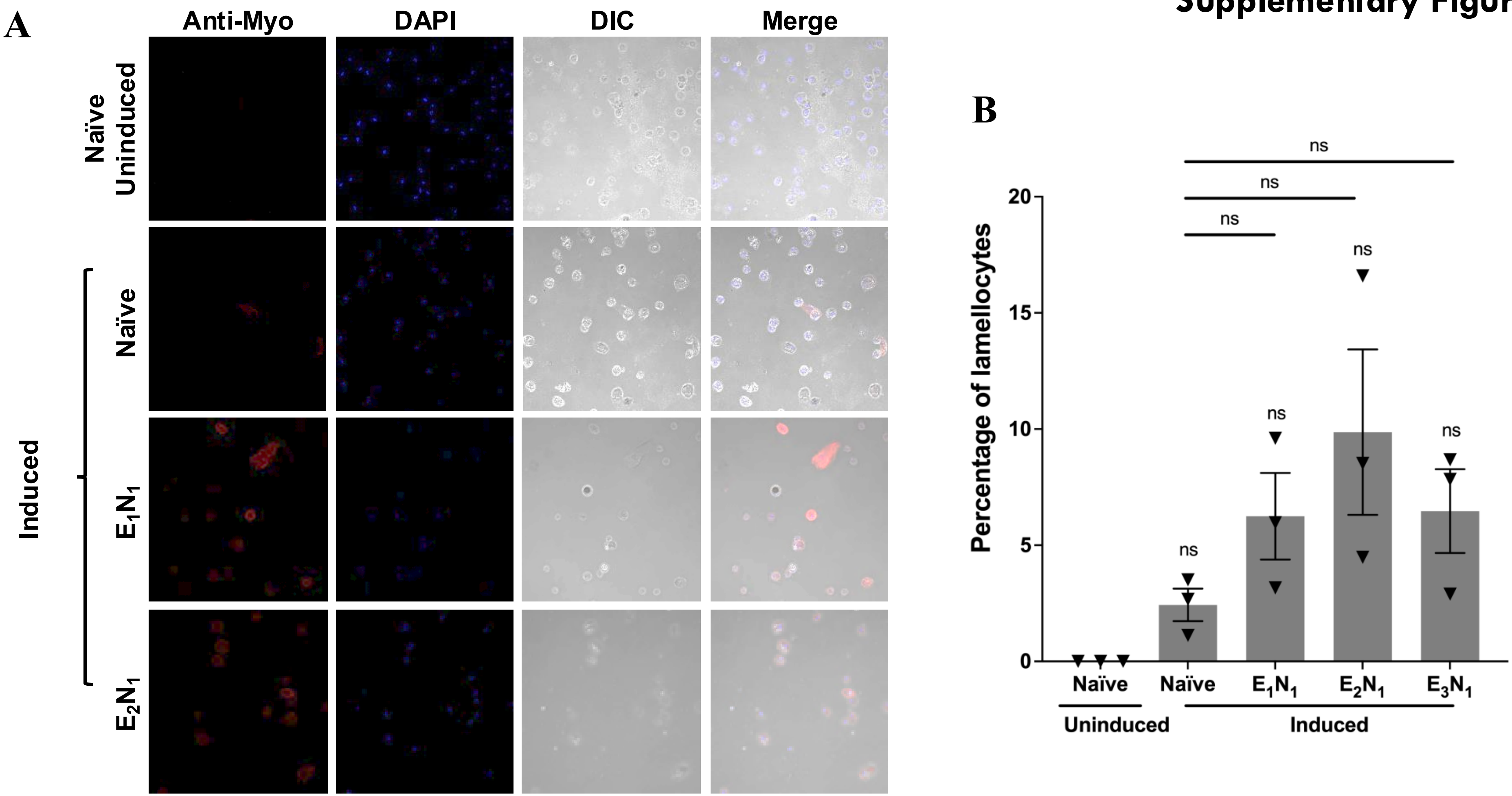

Figure S3. Cellular immune response to parasitic stress in progenies of female parents. (A) The panels depict images of circulatory hemolymph in third instar larvae from experienced female parents. In the first panel, hemocytes from naïve third instar larvae are shown, stained with anti-myospheroid (red) as a lamellocyte marker and DAPI (blue) for nuclei staining. The second panel shows hemocytes from third instar larvae that were mechanically induced to mimic a wasp attack at the second instar stage. The third panel presents hemocytes from induced larvae (E_1N_1) of an experienced female parent. The fourth panel shows hemocytes from induced larvae (E_2N_1) of an experienced female parent, whose own mother was also exposed to parasitic stress. Myospheroid-positive cells appear as fully developed lamellocytes (large and elongated) or as precursors committed to lamellocyte development, reflecting an enhanced cellular immune response. **(B)** The percentage of lamellocytes in the larval hemolymph, as depicted in the panels in (A), is shown here (see Supplementary Table S6). The experiment was conducted in three biological replicates, and error bars represent the standard error of the mean (SEM). Significance is indicated as ns for non-significant results ($p > 0.05$). P-values on the bars represent the significance between naïve uninduced and induced groups, while p-values on the connecting lines reflect the significance between naïve induced and other induced groups.
