# Supplementary material for: Multigenerational inheritance of parasitic stress memory in Drosophila melanogaster
Source: Environ Epigenet. 2025 Sep 4;11(1):dvaf023. doi: 10.1093/eep/dvaf023 (PMC12418946; doi:10.1093/eep/dvaf023)
Supplement: dvaf023_Supplemental_Files [file dvaf023_supplemental_files.zip › Supplementary Table S1.pdf]

Table S1: Biparental contribution to the parasitic stress memory (ten generations). Data related to Figure 1B

Generation	Control (One-time exposure)								Intergenerational Inheritance							p-value (Control vs Intergenerational
	Experience	Replicate	No. of larvae	No. of wasps	No. of escapees	Percentage of escapees	Mean	Mean normalized percentage of escapees	Experience	Replicate	No. of larvae	No. of wasps	Total escapees	Percentage of escapees	Mean normalized percentage of escapees	
F_1	N_1 or E_1	1	1000	592	21	2.1	1.9	1.11								NA
		2	1000	691	17	1.7		0.89								
F_2	N_2 or E_1	1	1000	524	36	3.6	3.45	1.04	E_2	1	579	343	54	9.33	2.70	0.03
		2	1000	651	33	3.3		0.96		2	690	374	71	10.29	2.98	
F_3	N_3 or E_1	1	1000	588	30	3	3	1.00	E_3	1	965	593	93	9.64	3.21	0.00
		2	1000	572	30	3		1.00		2	900	612	87	9.67	3.22	
F_4	N_4 or E_1	1	1000	553	29	2.9	2.85	1.02	E_4	1	1000	642	25	2.50	0.88	0.14
		2	1000	605	28	2.8		0.98		2	900	514	20	2.22	0.78	
F_5	N_5 or E_1	1	1000	409	27	2.7	2.6	1.04	E_5	1	1000	724	92	9.20	3.54	0.11
		2	1000	521	25	2.5		0.96		2	870	613	62	7.13	2.74	
F_6	N_6 or E_1	1	1000	600	21	2.1	2.2	0.95	E_6	1	1000	739	64	6.40	2.91	0.00
		2	1000	589	23	2.3		1.05		2	1000	733	66	6.60	3.00	
F_7	N_7 or E_1	1	1000	597	24	2.4	2.35	1.02	E_7	1	950	656	26	2.74	1.16	0.06
		2	1000	568	23	2.3		0.98		2	1000	768	27	2.70	1.15	
F_8	N_8 or E_1	1	1000	540	31	3.1	3	1.03	E_8	1	1000	768	56	5.60	1.87	0.22
		2	1000	603	29	2.9		0.97		2	1003	838	42	4.19	1.40	
F_9	N_9 or E_1	1	1000	560	26	2.6	2.75	0.95	E_9	1	1000	800	44	4.40	1.60	0.18
		2	1000	457	29	2.9		1.05		2	1000	783	59	5.90	2.15	
F_{10}	N_{10} or E_1	1	1000	613	17	1.7	1.6	1.06	E_{10}	1	1000	708	52	5.20	3.25	0.01
		2	1000	626	15	1.5		0.94		2	1000	754	49	4.90	3.06	
