# Supplementary material for: Multigenerational inheritance of parasitic stress memory in Drosophila melanogaster
Source: Environ Epigenet. 2025 Sep 4;11(1):dvaf023. doi: 10.1093/eep/dvaf023 (PMC12418946; doi:10.1093/eep/dvaf023)
Supplement: dvaf023_Supplemental_Files [file dvaf023_supplemental_files.zip › Supplementary Table S2.pdf]

Table S2: Biparental contribution to the parasitic stress memory (five generations). Data related to Figure 2A

Control (One-time exposure)

Generation	Experience	Replicate	No. of pupae	No. of wasps	No. of non-melanized escapees	No. of melanized escapees	Total escapees	Percentage of escapees	Mean	Mean normalized percentage of escapees
F_1	N_1 or E_1	1	386	321	5	7	12	3.11		1.06
		2	394	330	6	4	10	2.54	2.92	0.87
		3	385	324	4	8	12	3.12		1.07
F_2	N_2 or E_1	1	399	304	4	1	5	1.25		0.99
		2	157	146	2	2	4	2.55	1.27	2.01
		3	201	185	0	0	0	0.00		0.00
F_3	N_3 or E_1	1	313	300	2	5	7	2.24		1.09
		2	273	256	2	3	5	1.83	2.06	0.89
		3	331	317	1	6	7	2.11		1.03
F_4	N_4 or E_1	1	242	225	2	0	2	0.83		1.57
		2	401	364	3	0	3	0.75	0.52	1.43
		3	161	147	0	0	0	0.00		0.00
F_5	N_5 or E_1	1	196	192	0	0	0	0.00		0.00
		2	302	272	3	0	3	0.99	0.47	2.09
		3	233	221	0	1	1	0.43		0.91

Intergenerational Inheritance

Generation	Experience	Replicate	No. of pupae	No. of wasps	No. of non-melanized escapees	No. of melanized escapees	Total escapees	Percentage of escapees	Mean normalized percentage of escapees	Mean normalized percentage of escapees
F_2	E_2	1	332	257	10	4	14	4.22	3.33	
		2	511	421	1	21	22	4.31	3.40	0.05
		3	488	397	4	19	23	4.71	3.72	
F_3	E_3	1	607	510	8	11	19	3.13	1.52	
		2	502	456	5	7	12	2.39	1.16	0.29
		3	687	596	6	8	14	2.04	0.99	
F_4	E_4	1	395	364	10	2	12	3.04	5.79	
		2	546	477	9	3	12	2.20	4.19	0.00
		3	585	523	7	10	17	2.91	5.54	
F_5	E_5	1	540	476	8	6	14	2.59	5.47	
		2	536	461	8	6	14	2.61	5.51	0.00
		3	260	237	6	3	9	3.46	7.30	
