# Supplementary material for: Multigenerational inheritance of parasitic stress memory in Drosophila melanogaster
Source: Environ Epigenet. 2025 Sep 4;11(1):dvaf023. doi: 10.1093/eep/dvaf023 (PMC12418946; doi:10.1093/eep/dvaf023)
Supplement: dvaf023_Supplemental_Files [file dvaf023_supplemental_files.zip › Supplementary Table S3.pdf]

Table S3: Paternal contribution to the parasitic stress memory. Data related to Figure 2C and 2D

Generation	Control (One-time exposure)									
	Experience	Replicate	No. of pupae	No. of wasps	No. of non-melanized escapees	No. of melanized escapees	Total escapees	Percentage of escapees	Mean	Mean normalized percentage of escapees
F_1	N_1 or E_1	1	714	711	3	2	5	0.70	1.05	0.66
		2	643	635	0	4	4	0.62		0.59
		3	751	731	10	4	14	1.86		1.77
		4	777	770	4	3	7	0.90		0.85
		5	779	758	5	3	8	1.03		0.97
		6	577	570	3	4	7	1.21		1.15
F_2	N_2 or E_1	1	622	616	2	4	6	0.96	0.79	1.22
		2	755	748	5	2	7	0.93		1.18
		3	867	860	5	2	7	0.81		1.02
		4	621	617	3	1	4	0.64		0.82
		5	795	787	7	1	8	1.01		1.28
		6	780	777	3	0	3	0.38		0.49
F_3	N_3 or E_1	1	740	708	7	0	7	0.95	0.99	0.96
		2	716	702	3	0	3	0.42		0.42
		3	699	618	7	2	9	1.29		1.30
		4	716	669	7	0	7	0.98		0.99
		5	847	822	12	1	13	1.53		1.55
		6	785	700	6	0	6	0.76		0.77
F_4	N_4 or E_1	1	959	950	3	4	7	0.73	0.88	0.83
		2	864	811	3	5	8	0.93		1.05
		3	826	820	2	4	6	0.73		0.83
		4	845	834	3	7	10	1.18		1.35
		5	828	818	5	5	10	1.21		1.37
		6	792	785	1	3	4	0.51		0.57
F_5	N_5 or E_1	1	963	960	2	1	3	0.31	1.18	0.26
		2	999	983	10	6	16	1.60		1.36
		3	1319	1307	5	7	12	0.91		0.77
		4	976	969	0	7	7	0.72		0.61
		5	486	483	3	6	9	1.85		1.57
		6	538	529	9	0	9	1.67		1.42

Generation	Intergenerational Inheritance									
	Experience	Replicate	No. of pupae	No. of wasps	No. of non-melanized escapees	No. of melanized escapees	Total escapees	Percentage of escapees	Mean normalized percentage of escapees	p-value (Control vs Intergenerational)
F_2	E_2	1	630	611	9	7	16	2.54	3.22	0.01
		2	625	597	12	15	27	4.32	5.48	
		3	620	613	2	5	7	1.13	1.43	
		4	531	521	4	6	10	1.88	2.39	
		5	376	366	5	5	10	2.66	3.37	
		6	504	490	6	7	13	2.58	3.27	
F_3	E_3	1	1148	1110	13	25	38	3.31	3.35	<0.0001
		2	793	753	22	16	38	4.79	4.85	
		3	1028	990	17	19	36	3.50	3.54	
		4	1130	1090	29	11	40	3.54	3.58	
		5	1114	1075	23	16	39	3.50	3.54	
		6	1145	1105	26	14	40	3.49	3.54	
F_4	E_4	1	693	682	4	7	11	1.59	1.80	0.01
		2	797	781	12	4	16	2.01	2.28	
		3	921	894	14	13	27	2.93	3.33	
		4	806	880	11	15	26	3.23	3.67	
		5	883	859	17	7	24	2.72	3.09	
		6	683	675	2	7	9	1.32	1.50	
F_5	E_5	1	595	569	18	8	26	4.37	3.71	<0.0001
		2	489	470	10	9	19	3.89	3.30	
		3	472	451	9	12	21	4.45	3.78	
		4	612	583	14	15	29	4.74	4.02	
		5	560	536	13	11	24	4.29	3.64	
		6	733	701	12	20	32	4.37	3.71	

Generation	Transgenerational Inheritance									
	Experience	Replicate	No. of pupae	No. of wasps	No. of non-melanized escapees	No. of melanized escapees	Total escapees	Percentage of escapees	Mean normalized percentage of escapees	p-value (Control vs Transgenerational)
F_3	$E_1N_1E_1$	1	1096	1052	28	16	44	4.01	4.06	0.01
		2	1000	961	25	14	39	3.90	3.95	
		3	1033	1013	10	10	20	1.94	1.96	
		4	915	888	15	12	27	2.95	2.99	
		5	821	806	9	6	15	1.83	1.85	
F_4	$E_1N_2E_1$	1	458	444	3	11	14	3.06	3.47	0.00
		2	418	407	7	4	11	2.63	2.99	
		3	577	565	4	8	12	2.08	2.36	
		4	420	407	6	7	13	3.10	3.52	
		5	385	380	2	3	5	1.30	1.48	
		6	397	385	6	6	12	3.02	3.44	
