# Supplementary material for: Multigenerational inheritance of parasitic stress memory in Drosophila melanogaster
Source: Environ Epigenet. 2025 Sep 4;11(1):dvaf023. doi: 10.1093/eep/dvaf023 (PMC12418946; doi:10.1093/eep/dvaf023)
Supplement: dvaf023_Supplemental_Files [file dvaf023_supplemental_files.zip › Supplementary Tables S4.pdf]

Table S4: Maternal contribution to the parasitic stress memory. Data related to Figure S1

Control (One-time exposure)

Generation	Experience	Replicate	No. of pupae	No. of wasps	No. of non-melanized escapees	No. of melanized escapees	Total escapees	Percentage of escapees	Mean	Mean normalized percentage of escapees
F_1	N_1 or E_1	1	714	711	3	2	5	0.70	1.05	0.66
		2	643	635	0	4	4	0.62		0.59
		3	751	731	10	4	14	1.86		1.77
		4	777	770	4	3	7	0.90		0.85
		5	779	758	5	3	8	1.03		0.97
		6	577	570	3	4	7	1.21		1.15
F_2	N_2 or E_1	1	622	616	2	4	6	0.96	0.79	1.22
		2	755	748	5	2	7	0.93		1.18
		3	867	860	5	2	7	0.81		1.02
		4	621	617	3	1	4	0.64		0.82
		5	795	787	7	1	8	1.01		1.28
		6	780	777	3	0	3	0.38		0.49
F_3	N_3 or E_1	1	740	708	7	0	7	0.95	0.99	0.96
		2	716	702	3	0	3	0.42		0.42
		3	699	618	7	2	9	1.29		1.30
		4	716	669	7	0	7	0.98		0.99
		5	847	822	12	1	13	1.53		1.55
		6	785	700	6	0	6	0.76		0.77
F_4	N_4 or E_1	1	959	950	3	4	7	0.73	0.88	0.83
		2	864	811	3	5	8	0.93		1.05
		3	826	820	2	4	6	0.73		0.83
		4	845	834	3	7	10	1.18		1.35
		5	828	818	5	5	10	1.21		1.37
		6	792	785	1	3	4	0.51		0.57
F_5	N_5 or E_1	1	963	960	2	1	3	0.31	1.18	0.26
		2	999	983	10	6	16	1.60		1.36
		3	1319	1307	5	7	12	0.91		0.77
		4	976	969	0	7	7	0.72		0.61
		5	486	483	3	6	9	1.85		1.57
		6	538	529	9	0	9	1.67		1.42

Intergenerational Inheritance

Generation	Experience	Replicate	No. of pupae	No. of wasps	No. of non-melanized escapees	No. of melanized escapees	Total escapees	Percentage of escapees	Mean normalized percentage of escapees	p-value (Control vs Intergenerational)
F_2	E_2	1	474	465	5	4	9	1.90	2.41	0.03
		2	420	405	14	1	15	3.57	4.53	
		3	370	364	4	2	6	1.62	2.06	
		4	233	230	2	1	3	1.29	1.63	
		5	365	358	3	4	7	1.92	2.43	
F_3	E_3	1	377	383	1	3	4	1.06	1.07	0.28
		2	428	289	2	3	5	1.17	1.18	
		3	319	286	2	0	2	0.63	0.63	
		4	357	297	2	4	6	1.68	1.70	
		5	431	390	2	4	6	1.39	1.41	
		6	264	230	1	3	4	1.52	1.53	
F_4	E_4	1	420	348	2	3	5	1.19	1.35	0.33
		2	515	511	0	4	4	0.78	0.88	
		3	512	465	1	2	3	0.59	0.67	
		4	502	433	1	2	3	0.60	0.68	
		5	598	554	1	3	4	0.67	0.76	
		6	579	523	2	1	3	0.52	0.59	
F_5	E_5	1	666	655	4	7	11	1.65	1.40	0.37
		2	683	675	5	3	8	1.17	0.99	
		3	587	580	3	4	7	1.19	1.01	
		4	638	624	7	7	14	2.19	1.86	
		5	559	553	2	3	5	0.89	0.76	
		6	570	558	1	9	10	1.75	1.49	

Transgenerational Inheritance

Generation	Experience	Replicate	No. of pupae	No. of wasps	No. of non-melanized escapees	No. of melanized escapees	Total escapees	Percentage of escapees	Mean normalized percentage of escapees	p-value (Control vs Transgenerational)
F_3	$E_1N_1E_1$	1	413	407	2	2	4	0.97	0.98	0.48
		2	409	405	2	2	4	0.98	0.99	
		3	305	294	11	0	11	3.61	3.65	
		4	472	470	1	1	2	0.42	0.43	
		5	464	457	5	2	7	1.51	1.53	
		6	432	429	0	3	3	0.69	0.70	
F_4	$E_1N_2E_1$	1	415	412	1	2	3	0.72	0.82	0.06
		2	464	459	1	4	5	1.08	1.22	
		3	611	606	2	3	5	0.82	0.93	
		4	524	515	4	5	9	1.72	1.95	
		5	556	550	2	4	6	1.08	1.23	
		6	591	579	4	8	12	2.03	2.31	
