# Supplementary material for: Multigenerational inheritance of parasitic stress memory in Drosophila melanogaster
Source: Environ Epigenet. 2025 Sep 4;11(1):dvaf023. doi: 10.1093/eep/dvaf023 (PMC12418946; doi:10.1093/eep/dvaf023)
Supplement: dvaf023_Supplemental_Files [file dvaf023_supplemental_files.zip › Supplementary Tables S5.pdf]

Table S5: Effect of parasitic stress on fecundity. Data related to Figure S2

Mating	Replicate	Day 1	Day 2	Day 3	cundity per c	Mean	p-value
CS ☿ X CS ♂	1	8	8	14	10.00	21.11	NA
	2	22	29	29	26.67		
	3	23	20	20	21.00		
	4	22	23	20	21.67		
	5	26	23	21	23.33		
	6	22	29	21	24.00		
CS ☿ X E_1 ♂	1	37	13	5	18.33	18.78	0.55
	2	23	17	58	32.67		
	3	10	26	13	16.33		
	4	14	7	17	12.67		
	5	17	11	22	16.67		
	6	18	11	19	16.00		
E_1 ☿ X CS ♂	1	10	10	18	12.67	24.14	0.41
	2	29	29	23	27.00		
	3	20	47	22	29.67		
	4	20	42	27	29.67		
	5	17	25	25.5	22.50		
	6	23	24	23	23.33		
