# Supplementary material for: Multigenerational inheritance of parasitic stress memory in Drosophila melanogaster
Source: Environ Epigenet. 2025 Sep 4;11(1):dvaf023. doi: 10.1093/eep/dvaf023 (PMC12418946; doi:10.1093/eep/dvaf023)
Supplement: dvaf023_Supplemental_Files [file dvaf023_supplemental_files.zip › Supplementary Tables S6.pdf]

## Table S6: Cellular immune response to parasitic stress in the progenies. Data related to Figure 3B and S3B

| Treatment | Parents | Experience | Replicate | Total no. of hemocytes | No. of Mys+ cells | Percentage of Mys+ cells | Mean | p-value (Uninduced Naïve vs Induced Experienced | p-value (Induced Naïve vs Induced Experienced |
|---|---|---|---|---|---|---|---|---|---|
| Uninduced | **Naïve** | None | 1 | 2903 | 0 | 0.00 | 0.00 | NA | NA |
| | | | 2 | 3056 | 0 | 0.00 | | | |
| | | | 3 | 3318 | 0 | 0.00 | | | |
| Induced | | None | 1 | 1983 | 53 | 2.67 | 2.43 | 0.07 | NA |
| | | | 2 | 1996 | 70 | 3.51 | | | |
| | | | 3 | 2063 | 23 | 1.11 | | | |
| | **Experienced males** | $E_1N_1$ | 1 | 1887 | 277 | 14.68 | 18.11 | 0.01 | 0.01 |
| | | | 2 | 7553 | 1402 | 18.56 | | | |
| | | | 3 | 7227 | 1524 | 21.09 | | | |
| | | $E_2N_1$ | 1 | 6643 | 1543 | 23.23 | 28.51 | 0.02 | 0.02 |
| | | | 2 | 2018 | 731 | 36.22 | | | |
| | | | 3 | 1630 | 425 | 26.07 | | | |
| | | $E_3N_1$ | 1 | 1198 | 119 | 9.93 | 13.89 | 0.14 | 0.19 |
| | | | 2 | 2967 | 758 | 25.55 | | | |
| | | | 3 | 2201 | 136 | 6.18 | | | |
| | **Experienced females** | $E_1N_1$ | 1 | 3228 | 310 | 9.60 | 6.25 | 0.08 | 0.17 |
| | | | 2 | 4542 | 271 | 5.97 | | | |
| | | | 3 | 1484 | 47 | 3.17 | | | |
| | | $E_2N_1$ | 1 | 2019 | 335 | 16.59 | 9.87 | 0.11 | 0.17 |
| | | | 2 | 3367 | 287 | 8.52 | | | |
| | | | 3 | 2990 | 134 | 4.48 | | | |
| | | $E_3N_1$ | 1 | 2489 | 72 | 2.89 | 6.47 | 0.07 | 0.14 |
| | | | 2 | 2972 | 258 | 8.68 | | | |
| | | | 3 | 1711 | 134 | 7.83 | | | |
